# The Differences in Local Translatome across Distinct Neuron Types Is Mediated by Both Baseline Cellular Differences and Post-transcriptional Mechanisms

**DOI:** 10.1523/ENEURO.0320-18.2018

**Published:** 2019-02-04

**Authors:** Rebecca Ouwenga, Allison M. Lake, Shivani Aryal, Tomas Lagunas, Joseph D. Dougherty

**Affiliations:** 1 Division of Biology and Biomedical Sciences, Washington University School of Medicine, St. Louis, Missouri 63110; 2 Department of Genetics, Washington University School of Medicine, St. Louis, Missouri 63110; 3 Department of Psychiatry, Washington University School of Medicine, St. Louis, Missouri 63110; 4 Hope Center for Neurological Disorders, Washington University School of Medicine, St. Louis, Missouri 63110

**Keywords:** GABAergic interneurons, layer 5 pyramidal neurons, local translation, synaptoneurosomes, SynapTRAP, TRAP

## Abstract

Local translation in neurites is a phenomenon that enhances the spatial segregation of proteins and their functions away from the cell body, yet it is unclear how local translation varies across neuronal cell types. Further, it is unclear whether differences in local translation across cell types simply reflect differences in transcription or whether there is also a cell type-specific post-transcriptional regulation of the location and translation of specific mRNAs. Most of the mRNAs discovered as being locally translated have been identified from hippocampal neurons because their laminar organization facilitates neurite-specific dissection and microscopy methods. Given the diversity of neurons across the brain, studies have not yet analyzed how locally translated mRNAs differ across cell types. Here, we used the SynapTRAP method to harvest two broad cell types in the mouse forebrain: GABAergic neurons and layer 5 projection neurons. While some transcripts overlap, the majority of the local translatome is not shared across these cell types. In addition to differences driven by baseline expression levels, some transcripts also exhibit cell type-specific post-transcriptional regulation. Finally, we provide evidence that GABAergic neurons specifically localize mRNAs for peptide neurotransmitters, including somatostatin and cortistatin, suggesting localized production of these key signaling molecules in the neurites of GABAergic neurons. Overall, this work suggests that differences in local translation in neurites across neuronal cell types are poised to contribute substantially to the heterogeneity in neuronal phenotypes.

## Significance Statement

All RNAs are generated in the nucleus, but in neurons some of these RNAs are shuttled for local protein production in neurites toward synapses. There are many types of neurons that express different complements of receptors and perform different functions. However, there has not yet been a direct comparison of the ribosome-bound transcripts in neurites across cells. Here, we identify and define differences in ribosome-bound RNAs isolated from neurites of two contrasting types of neurons. Some of these differences are due to the neurons not creating the RNA at baseline and some are differences in RNA localization or ribosome binding in neurites. We also identified RNAs for key neurotransmitter proteins that had not been previously described as produced in neurites, suggesting these may be locally produced.

## Introduction

Neurons have the ability to localize specific RNAs in neurites, and local translation near synapses has been shown to be essential to the kind of synaptic alterations that are thought to underlie many functions from the formation of neurites to learning and memory. Evidence for localized translation in neurites was first observed in 1982 (Steward and Levy, 1982), but only recently have advances in molecular techniques enabled the identification of locally translated mRNAs in a high-throughput manner. These studies have identified many candidate mRNAs for local translation in neurites *in vivo*. However, a substantial fraction of prior studies has focused on hippocampal neurons because their spatially separated dendritic and somatic layers facilitate analysis (Van Driesche and Martin, 2018). In contrast, most other neuron types in the brain have highly interwoven neurites such that physical dissection is unable to harvest a sample enriched for the processes of specific cells. Thus, there have been no direct comparisons of how local translation in neurites may differ across distinct cell types of neurons in the brain.

Cell types have classically been defined by differences in location, morphology, neurotransmitter usage, and function. In the last decade, it has become clear that there are corresponding whole-cell transcriptional differences between cell types (Heiman et al., 2008; Dougherty, 2013; Xu et al., 2014; Zhang et al., 2014). However, to what extent these cells also have differences in the subcellular localized translation of transcripts is unclear. Comparison of the vast, branched arbor of a Purkinje neuron with the short, clawed dendrites of a granule cell highlights the remarkable diversity in neurites even just within the cerebellum. Likewise, inhibitory and excitatory neurons differ in both function and morphology within the forebrain. Cortical pyramidal neurons are large, excitatory neurons with long apical dendrites that extend to the upper layers of cortex and axons projecting to distal brain structures. Cortical inhibitory interneurons often have much shorter neurites that project onto neighboring cells (Wonders and Anderson, 2006; DeFelipe et al., 2013).

Distinct profiles in local translation could be a cause and a consequence of the large variety of neuronal morphologies and functions across the brain. Therefore, we hypothesize that fundamental morphologic and functional differences across neuronal types will be reflected in clear distinctions in local translational profiles as well. Furthermore, distinctive local translational profiles could simply reflect either transcriptional differences or differences in the post-transcriptional regulation of mRNA localization or stability. For example, on one hand, only one cell type might express a transcript, and thus only that cell type could possibly translate it locally. On the other hand, two different cell types might express the same transcript, yet in one transcript localization is altered through the recognition of a motif in the 3´ UTR by a cell type-specific RNA binding protein (RNABP) that shuttles mRNAs to neurites or binds a secondary structure of mRNA that can alter the rate at which they degrade (Andreassi and Riccio, 2009; Patel et al., 2012). Thus, the differential expression of RNABPs between cell types could plausibly result in distinct profiles of locally translated RNAs. The identification of how and why locally translated transcripts differ across cell types could further define pathways that underlie morphologic and functional differences.

Previously, we developed a derivative of the translating ribosome affinity purification (TRAP) method, SynapTRAP (ST), to enable the enrichment of ribosome-bound transcripts from the processes of genetically targeted cell types of the mouse brain (Dougherty, 2017; Ouwenga et al., 2017). Here, we used two TRAP mouse lines to determine whether local translation, as assessed by SynapTRAP, is distinct across different cell types of neurons. Specifically, we compared layer 5 pyramidal neurons of the cortex to GABAergic neurons of the forebrain. These neuronal types were chosen as they differ in prior translational profiles, morphologic features, and functions. We also sought to determine whether the differences in the local translatome are due to baseline, likely transcriptional, differences or to post-transcriptional regulation. We found that the majority of the differences in local translation were likely driven by baseline variations, but there is a measurable role for post-transcriptional regulation as well. Finally, we determine that two of these cell type-specific locally translated mRNAs encode for the peptide transmitters cortistatin and somatostatin, suggesting local production of peptide neurotransmitters.

## Materials and Methods

### Animals

All procedures were performed in accordance with the guidelines of the Institutional Animal Care and Use Committee. Mice were maintained in standard housing conditions with food and water provided *ad libitum*. RNA collection used a Cre-dependent TRAP reporter mouse B6.129S4-Gt(ROSA)^26Sortm1(CAG-EGFP/Rpl10a,-birA)Wtp^/J (catalog #22367, The Jackson Laboratory; RRID:IMSR_JAX:022367; Zhou et al., 2013) that were bred to two well characterized Cre lines: Tg(RBP4-cre)^KL100Gsat/Mmcd^ (RRID:MMRRC_037128-UCD; Beltramo et al., 2013) and Slc32a1^tm2(cre)Lowl^/J (catalog #16962, The Jackson Laboratory; RRID:IMSR_JAX:016962; Vong et al., 2011) then genotyped for the presence of the TRAP construct and Cre. Mice positive for both a Cre and TRAP construct are referred to as RBP4-TRAP and vesicular GABA transporter (VGAT)-TRAP mice, respectively.

### Experimental design and statistical analysis

All statistical tests are reported in the subsection of the Materials and Methods describing each experiment. The design, sample sizes, intermediate values, and results can be found in the legend of each figure in which they are represented. The SynapTRAP preparations and quantitative PCR (qPCR) were performed with both sexes pooled. *In situ* hybridization (ISH) replication used an equal number of male and female mice for each probe. All data are available on the NCBI Genome Expression Omnibus web site (accession #GSE121162).

### SynapTRAP, library preparation, and RNA sequencing

Five replicates of RBP4-TRAP and VGAT-TRAP were harvested by rapid forebrain dissection at 21 d postbirth, as described previously (Westmark et al., 2011; Ouwenga et al., 2017). Each replicate contained a pool of two to three forebrains of both sexes, as available. Four samples were collected from each replicate in parallel, as follows: whole-cell homogenate (WCH) was RNA isolated from an aliquot of the initial homogenization of the tissue, and TRAP was the capture of GFP-tagged ribosomes from an aliquot of WCH. RNA isolated from a fraction of the WCH subjected to synaptoneurosomal fractionation (SNF), and ST is TRAP performed on the SNF, as described previously (Ouwenga et al., 2017). RNA concentration for all was measured using a Nanodrop and diluted to <5 ng/µl before being assessed for quality and concentration using an Agilent TapeStation 4200.

Library preparation was performed with 30 ng of total RNA from each sample. Double-stranded cDNA was prepared using the SMARTer Ultra Low RNA kit for Illumina Sequencing (catalog #634936, Clontech) per manufacturer protocol. cDNA was fragmented using a Covaris E220 Sonicator using peak incident power of 18, a duty factor of 20%, 50 cycles/burst, and a time of 120 s at 18ºC. cDNA was blunt ended, had an A base added to the 3´ ends, and then had Illumina sequencing adapters ligated to the ends. Ligated fragments were then amplified for 13 cycles using primers incorporating unique index tags. Fragments were sequenced on an Illumina HiSeq-3000 sequencing device using single reads extending 50 bases to a depth of 14.4–22 million reads per sample.

### RNA sequencing data quality control, and processing

RNA sequencing (RNA-seq) reads were aligned to the Ensembl top-level assembly with STAR version 2.0.4b (RRID:SCR_015899; Dobin et al., 2013). Gene counts were derived from the number of uniquely aligned unambiguous reads by Subread:featureCount version 1.4.5. Sequencing performance was assessed with RSeQC version 2.3 (RRID:SCR_005275; L. Wang et al., 2012) for the total number of aligned reads, the total number of uniquely aligned reads, the genes and transcripts detected, the ribosomal fraction, the known junction saturation, and the read distribution over known gene models. Gene-level counts were then imported into the R/Bioconductor package EdgeR (RRID:SCR_012802; Robinson et al., 2010). Mitochondrial ribosomal RNA (rRNA), tRNA, mitochondrial and remaining eukaryotic rRNA reads were excluded, as were genes without at least 0.5 cpm in at least three samples for creation of local and “somatic” candidate lists, or in at least three TRAP or ST samples for the later direct differential expression comparisons between the two lines. Counts were then normalized to a final number of counts per million based on the final library sizes.

For quality control, performance of the replicate samples was assessed with a Spearman correlation matrix and multidimensional scaling plots and RNA-seq results were verified as reproducible: technical replicates clustered together both in hierarchical clustering based on the highest 5000 transcripts by counts per million and in multidimensional scaling.

Then, gene-level performance was assessed with plots of the residual SD of every gene to their average log count with a robustly fitted trend line of the residuals. Generalized linear models using the negative binomial were then created to test for gene-level differential expression with EdgeR (RRID:SCR_012802; Robinson et al., 2010), using the contrasts described in the three sections below. Differentially expressed genes and transcripts were then filtered for false discovery rate (FDR)-adjusted *p* values ≤0.05, except where noted in the text.

### Defining the translational profile of layer 5 neurons and GABAergic neurons

All differential expression analysis was performed using single-variable generalized linear model approaches implemented in EdgeR, with the following grouping variables: RBP4_ST; VGAT_ST; RBP4_TRAP; VGAT_TRAP; RBP4_WCH; and VGAT_WCH.

**Figure 1.**
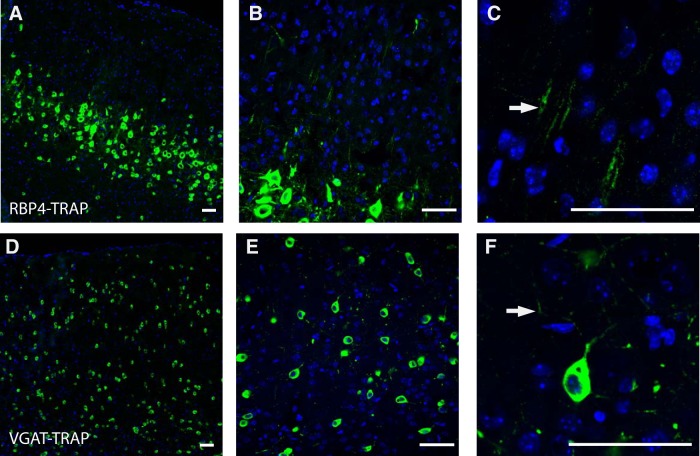
Immunofluorescence of RBP4-TRAP and VGAT-TRAP lines shows expected cellular expression patterns and localization of ribosomal protein L10a-GFP fusion to neurites. ***A***, RBP4-driven Cre line expresses the TRAP construct, designed to tag ribosomes with GFP, in layer 5 pyramidal neurons (10×). ***B***, Labeling extends into primary dendrites that continue into the upper layers of the cortex (40×). ***C***, An example of a RBP4-TRAP dendrite with GFP-tagged ribosomal proteins (arrow). ***D***, VGAT-driven Cre line expresses the TRAP construct in pattern consistent with interneurons of the cortex (10×). ***E***, Images at a higher magnification (40×) highlight that the GFP-tagged ribosomal proteins localize in neurites. ***F***, An example of the neurites of a single VGAT-TRAP neuron with GFP-tagged ribosomal proteins (arrow). Green, GFP; blue, DAPI nuclear stain. Scale bars, 50 μm.

**Figure 2.**
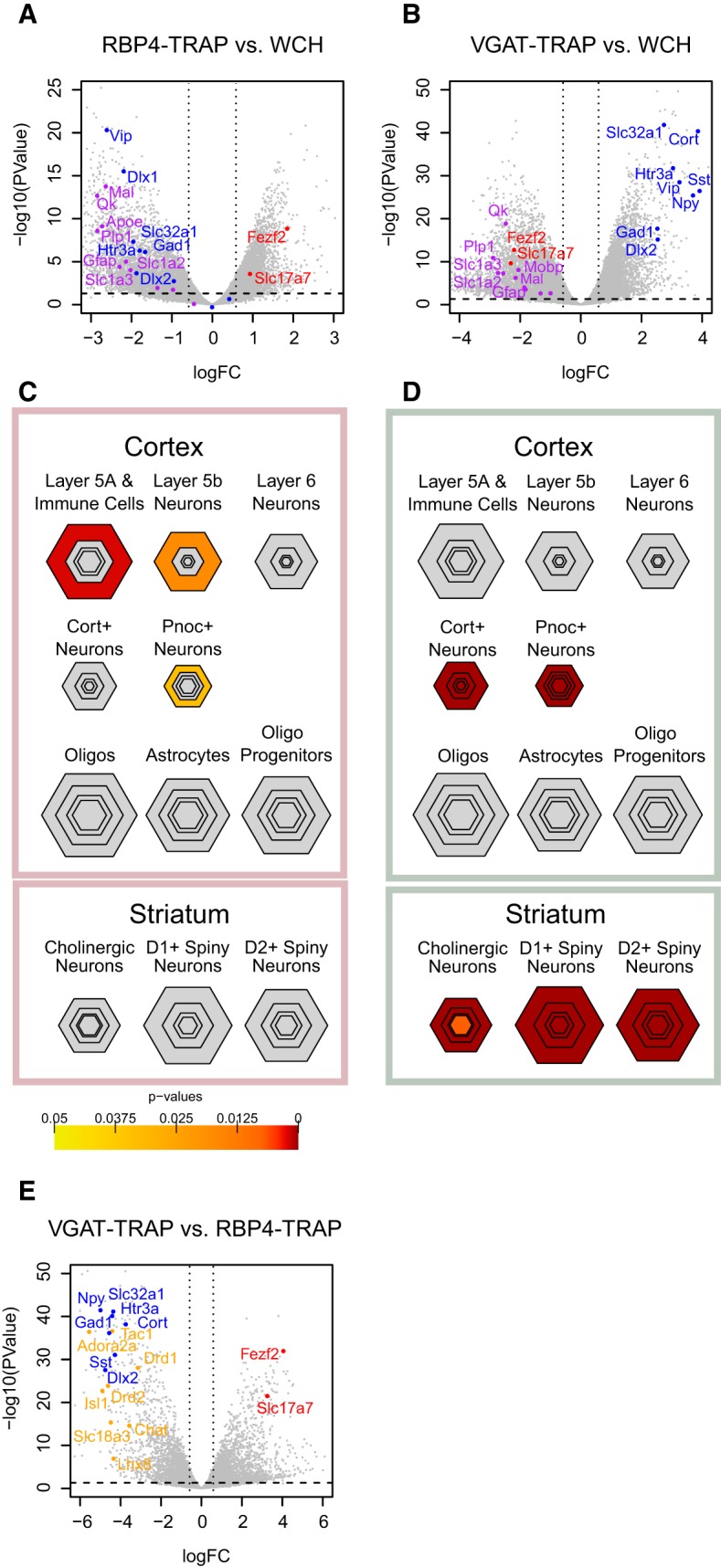
TRAP enriches ribosome-bound mRNA from cortical projection neurons and GABAergic forebrain populations. ***A***, A volcano plot illustrates thousands of transcripts that are enriched by RBP4-TRAP when compared with cortical RNA (WCH). This includes known layer 5 transcripts (red) including the vesicular glutamate transporter 1 (*Slc17a7*) and the transcription factor *Fezf2*. The sample is relatively depleted for glial genes (purple) and markers of interneurons (blue). The horizontal dashed line demarcates *p* < 0.05, and vertical lines down 1.5-fold show enrichment or depletion. ***B***, A volcano plot illustrates that thousands of transcripts are enriched in VGAT-TRAP compared with WCH, including known markers of interneurons such as VGAT (*Slc32a1*), Gad, *Npy*, *Sst*, *Cort*, and *Dlx2*. Glial and layer 5 transcripts are depleted. ***C***, CSEA analysis of the top 200 genes enriched in RBP4-TRAP over WCH shows significant overlap with previously defined layer 5 expressed genes. Hexagons represent gene lists enriched to each cell type, with smaller hexagons representing smaller and more stringent gene lists. These are color coded by the significance of Fisher exact test results on overlap with RBP4-TRAP data. ***D***, CSEA analysis of top 200 genes enriched in VGAT-TRAP over WCH significantly overlap with previously defined interneuron-expressed genes (Pnoc^+^ and Cort^+^ TRAP lines), as well as genes expressed in striatal GABAergic cells (D1^+^, D2^+^, and ChAT neurons). ***E***, Volcano plot of a direct comparison of VGAT-TRAP with RBP4-TRAP demonstrates even more robust enrichment of layer 5 transcripts and makers of GABAergic cells in cortex (blue) and confirms the expression of markers of GABAergic neurons of striatum (orange). Figure 2-1: DE results between TRAP and WCH of RBP4-TRAP. Figure 2-2: DE results between TRAP and WCH of VGAT-TRAP. Figure 2-3: DE results between TRAP RBP4-TRAP and TRAP VGAT-TRAP.

**Figure 3.**
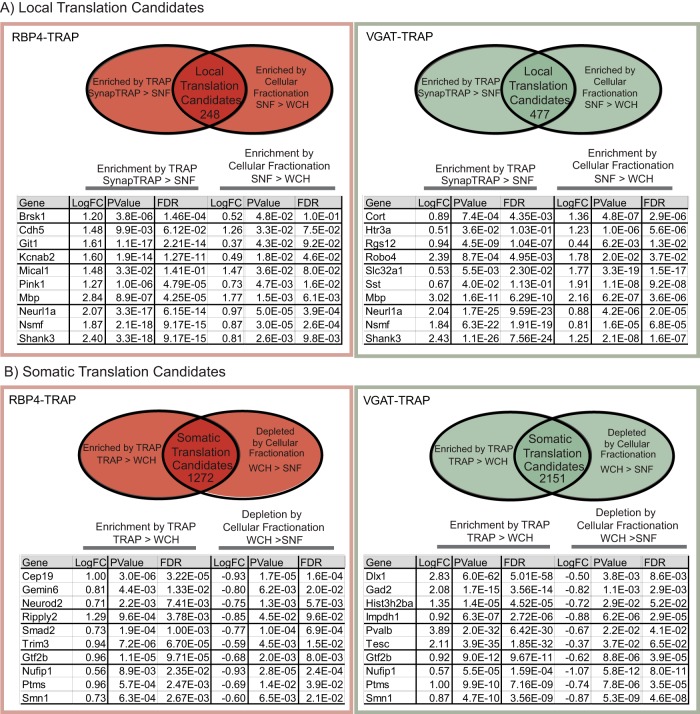
SynapTRAP identifies local translation candidate transcripts for both RBP4 and VGAT-TRAP. ***A***, Tables of 10 representative local translation candidate transcripts from each of the cell types and Venn diagrams illustrating the analysis conditions used to generate each list of candidates. ***B***, Tables of 10 representative somatic candidates from each of the cell types and Venn diagrams illustrating the analysis conditions used to generate each list of candidates. Figure 3-1: Table of RBP4 Local Translation Candidates. Figure 3-2: Table of VGAT Local Translation Candidates. Figure 3-3: Table of RBP4 Somatic Translation Candidates. Figure 3-4: Table of VGAT Somatic Translation Candidates.

The expression of TRAP samples was compared with the corresponding WCH samples to define transcripts significantly enriched in each type of neuron compared with overall forebrain gene expression using EdgeR (contrasts: RBP4_TRAP vs RBP4_WCH and VGAT_TRAP vs VGAT_WCH). These form the basis of Figure 2-1, and Figure 2-2. Next, to identify differences between the neuronal cell types, each TRAP sample was also directly compared (Fig. 2-3; contrast: VGAT_TRAP vs RBP4_TRAP).

10.1523/ENEURO.0320-18.2018.f2-1 Figure 2-1 DE results between TRAP and WCH of RBP4-TRAP. Table of differential expression results between TRAP and WCH of RBP4-TRAP. Genes with transcripts enriched in RBP4-TRAP cells over background. Gene_id, Ensembl gene ID; Entrez gene, enterez gene name; gene_name, corresponding gene symbol; Chr, chromosome, with EdgeR output for each comparison [including logFC (log base twofold change)]; logcpm, counts per million in log scale; LR, likelihood ratio. Download Figure 2-1, XLS file.

10.1523/ENEURO.0320-18.2018.f2-2 Figure 2-2 Table of differential expression results between TRAP and WCH of VGAT-TRAP. Genes with transcripts enriched in VGAT-TRAP cells over background. Column names are as in Figure 2-1. Download Figure 2-2, XLS file.

10.1523/ENEURO.0320-18.2018.f2-3 Figure 2-3 DE results between TRAP RBP4-TRAP and TRAP VGAT-TRAP. Table of differential expression results between RBP4-TRAP and TRAP VGAT-TRAP. Genes with transcripts enriched in each cell type compared with each other. Column names are as in Figure 2-1. Download Figure 2-3, XLS file.

### Defining the local and somatic translation candidates in each neuron type

**Figure 4.**
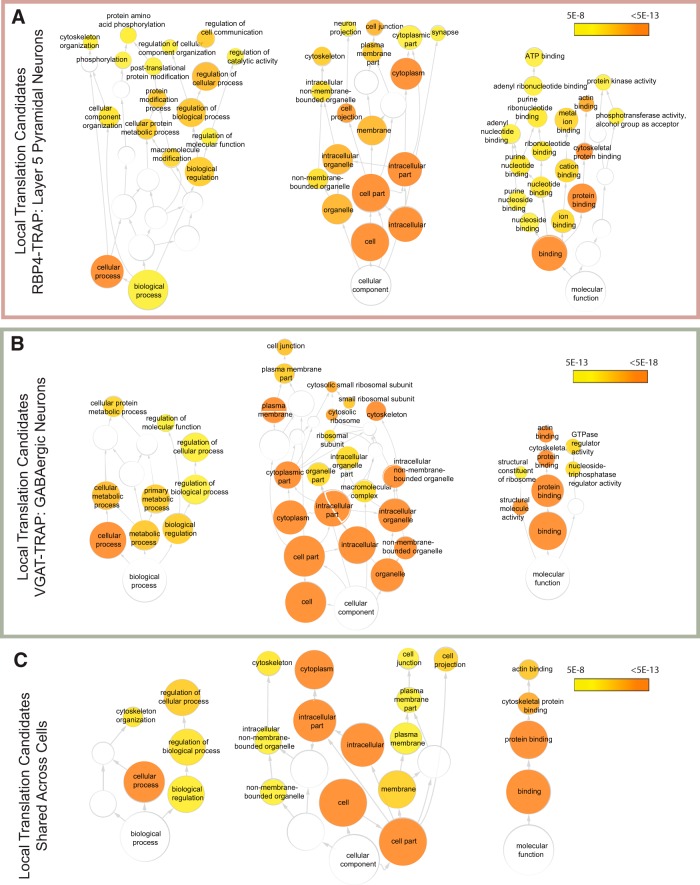
Pathway analysis of local translation candidates. ***A***, A gene ontologies pathway analysis of local translation candidates from the RBP4-TRAP data representing pyramidal neurons reveals significant enrichment of a variety of functional gene categories. Colored nodes represent significant categories and are organized hierarchically from broadest categories (bottom of each tree) to most specific. ***B***, A gene ontologies analysis of local translation candidates from the VGAT-TRAP mouse line, representing GABAergic neurons reveals enrichment in cytoskeletal elements and ribosomal proteins, among other categories. ***C***, A gene ontologies analysis of those transcripts found as local translation candidates in both VGAT-TRAP and RBP4-TRAP mouse lines (intersect) reveals a common theme of cytoskeletal elements. The color key indicates the significance of hypergeometic testing after Benjamini–Hochberg multiple testing correction. Only categories with *p* < 10E-8 (***A***, ***C***) or *p* < 10E-13 are shown (***B***). Figure 4-1: GO analysis results of RBP4 local translation candidates with a significance cutoff *p* = 5E-8. Figure 4-2: GO analysis results of VGAT local translation candidates with a significance cutoff *p* = 5E-8. Figure 4-3: GO analysis results of shared local translation candidates with a significance cutoff *p* = 5E-8. Figure 4-4: GO analysis results of RBP4 TRAP vs WCH enriched transcripts with a significance cutoff *p* = 5E-8. Figure 4-5: GO analysis results of VGAT TRAP vs WCH enriched transcripts with a significance cutoff *p* = 5E-8.

Local translation candidates [(cpm_SynapTRAP_ > cpm_SNF_) ∩ (cpm_SNF_ > cpm_WCH_)] were identified as those enriched by both SNF and by TRAP using a single-variable generalized linear model. Somatic transition candidates [(cpm_WCH_ > cpm_SNF_) ∩ (cpm_TRAP_ > cpm_WCH_)] were identified as those enriched by TRAP but depleted by cellular fractionation. These are as described in the study by Ouwenga et al. (2017) and are reported in Figure 3-1, Figure 3-2, Figure 3-3, and Figure 3-4.

10.1523/ENEURO.0320-18.2018.f3-1 Figure 3-1 Table of RBP4 local translation candidates. Full table of RBP4 local translation candidates. Table of transcripts enriched by both cellular fractionation and by TRAP on RBP4-TRAP mice. Column names are as in Figure 2-1. Two sets of EdgeR analyses included, byColumn refers to SNF > WCH comparison. byTRAP refers to TRAP > WCH comparison. Download Figure 3-1, XLS file.

10.1523/ENEURO.0320-18.2018.f3-2 Figure 3-2 Table of VGAT local translation candidates. Full table of VGAT local translation candidates. Table of transcripts enriched by both cellular fractionation and by TRAP in VGAT-TRAP mice. Column names are as in Figure 2-1. Download Figure 3-2, XLS file.

10.1523/ENEURO.0320-18.2018.f3-3 Figure 3-3 Table of RBP4 somatic translation candidates. Full table of RBP4 somatic translation candidates. Table of transcripts enriched by TRAP but depleted by cellular fractionation in RBP4-TRAP mice. Column names are as in Figure 2-1. Download Figure 3-3, XLS file.

10.1523/ENEURO.0320-18.2018.f3-4 Figure 3-4 Table of VGAT somatic translation candidates. Full table of VGAT somatic translation candidates. Table of transcripts enriched by TRAP but depleted by cellular fractionation in VGAT-TRAP mice. Column names are as in Figure 2-1. Download Figure 3-4, XLS file.

### Defining differential translation candidates between neuron types

The desired comparisons between groups were achieved using the following contrasts: WCH = RBP4_WCH − VGAT_WCH; ST_RBP4vVGAT = RBP4_ST − VGAT_ST; interaction = (RBP4_ST − VGAT_ST) − (RBP4_TRAP − VGAT_TRAP).

We first confirmed that only a small number of genes was differentially expressed in the WCH contrasts between the two lines. These were excluded from all differential expression candidate lists.

Next, the “ST_RBP4vVGAT” contrast represents a direct comparison between the ST samples of the two cell types. The significant candidates are reported in Figure 6-1, and Figure 6-2. Finally, to identify post-transcriptional regulation, we used an “Interaction” comparison to represent the difference in the effect cell type between the two cellular compartments (TRAP compared with ST). This is analogous to an interaction effect in a two-way ANOVA and is reported in Figure 9-1.

### Gene ontology and cell type-specific expression analysis

Gene ontologies pathway analysis was conducted with the BINGO (3.0.3) plugin for Cytoscape 2.8.2 (Maere et al., 2005). A hypergeometric test with Benjamini–Hochberg multiple testing correction was implemented to detect overrepresented categories from GO_MF, GO_BP, and GO_CC using a cutoff of *p* = 5E-8. Results in Figure 4-1, Figure 4-2, Figure 4-3, Figure 7-1, and Figure 7-2.

10.1523/ENEURO.0320-18.2018.f4-1 Figure 4-1 GO analysis results of RBP4 local translation candidates with a significance cutoff, *p* = 5E-8. Full table of GO analysis results of RBP4 local translation candidates with a significance cutoff, *p* = 5E-8. Column headers are generated by BINGO and include *GO ID* (Gene Ontologies Category number), *Description* (provided by GO), *p* value of hypergeometic, test, corr p-val (*p* value after Benjamin-Hochberg correction), *cluster freq* (number of GO genes in the local translation list), *total freq* (number of genes in the GO term list compared with all annotated genes), and *genes* (a list of the genes in the intersection). Download Figure 4-1, XLS file.

10.1523/ENEURO.0320-18.2018.f4-2 Figure 4-2 GO analysis results of VGAT local translation candidates with a significance cutoff, *p* = 5E-8. Full table of GO analysis results of VGAT local translation candidates with a significance cutoff, *p* = 5E-8. Column headers are as in Figure 4-1. Download Figure 4-2, XLS file.

10.1523/ENEURO.0320-18.2018.f4-3 Figure 4-3 GO analysis results of shared local translation candidates with a significance cutoff, *p* = 5E-8. Full table of GO analysis results of shared local translation candidates with a significance cutoff, *p* = 5E-8. Column headers are as in Figure 4-1. Download Figure 4-3, XLS file.

Cell type-specific expression analysis (CSEA) was conducted as described previously (Xu et al., 2014), using the top 200 genes with FDR <0.05, sorted by log fold change, enriched in TRAP sample compared with the corresponding WCH (see Fig. 2*C*,*D*).

### Sequence feature analysis

The 3´ UTRs of the local translation candidates were downloaded through Biomart (RRID:SCR_002987; Smedley et al., 2015). The longest available UTR sequence for each candidate was selected. To identify enriched motifs (MEME Suites), these 3´ UTRs were input to MEME Suites version 4.12.0 (RRID:SCR_001783; Bailey et al., 2009). For a comparison group, the longest available UTRs were also downloaded for the somatic translation candidates (top 500 by combined absolute value of the log fold change) from the corresponding cell type, and these were used as background controls for the identification of motifs in the local translation candidates. In a second analysis focusing on the differentially translated candidates between cell types, for the UTR of each cell type, the 3´ UTR sequences of the alternate cell type were used as controls for the identification of known motifs [AME (Analysis of Motif Enrichment)].

### Immunofluorescence

Brains were harvested from postnatal day 21 mice and fixed for 48 h in 4% paraformaldehyde followed by 48 h in 30% sucrose in 1× PBS. Brain was then cut down the midline before freezing in OCT compound (optimum cutting temperature compound; catalog #23-730-571, Thermo Fisher Scientific). A cryostat was used for slide mounting 10 μm sagittal sections of brain tissue. Slides were stored at −80°C.

Slides were incubated in a blocking solution (PBS, 5% donkey serum, 0.1% Triton-X 100) for 1 h in a humidified chamber at room temperature, then with chicken anti-GFP primary antibody (1:1000; RRID:AB_10000240) in blocking solution overnight in humidified chamber at 4°C. Following washes in PBS, slides were incubated in donkey anti-chicken Alexa Fluor-488 secondary antibody (diluted 1:400 in blocking solution). Slides were washed with PBS, incubated with DAPI, washed again, and mounted with Prolong Gold.

### Fluorescence ISH

High titer of a virus expressing a yellow fluorescent protein (YFP)-tagged membrane-localized protein (channelrhodopsin), serotyped as AAV5 [adeno-associated virus 5; AAV5-EF1a-DIO-hChR2(H134R)-EYFP], was obtained from the Hope Center Viral Vector Core (Washington University School of Medicine). One-day-old pups from the two Cre lines were injected with the virus to produce sparse Cre-dependent labeling of neurite membranes. At 21 d, the animals underwent a 4% paraformaldehyde transcardial perfusion, 12 h incubation in 15% sucrose in PBS, and 12 h incubation in 30% sucrose in PBS at 4°C. Coronal sections (18 μm) were cut onto slides with a cryostat, and these were postfixed in 4% paraformaldehyde. Slides were hybridized at 63°C with a 100 ng Dig-labeled antisense RNA probe created with T7 polymerase (catalog #P2075, Promega), from PCR products using primer sequences from the Allen Brain Atlas (Lein et al., 2007), and DIG RNA Labeling Mix (catalog #11277073910, Roche) according to the manufacturer protocol. cDNA was created using Superscript 3 and random hexamer priming (catalog #18080093, Thermo Fisher Scientific). Probe detection was performed using Sheep Anti-Dig-POD (catalog #11207733910, Roche) followed by Tyramide Signal Amplification Cyanine 3 Tyramide (catalog #NEL704A001KT, PerkinElmer). Post-ISH, slides underwent an immunofluorescence labeling as described above. Samples were imaged on a Zeiss Imager.Z2 confocal microscope at 1048 × 1048 pixel resolution at constant settings across probes. For each probe, three slices from each of the two RBP4-CRE mice and two VGAT-CRE mice (male and female) were imaged for YFP-positive neurites using a 40× oil lens.

Images were quantified using ImageJ software by macros that were consistent across probes. First, each channel was converted to a black and white image using a threshold on brightness. Using the image calculator, the area of NeuN and DAPI were subtracted from the YFP channel to mask the soma area of the neurons. This area was quantified and used as a YFP-neurite area. Overlapping puncta from the Cy3 ISH signal channel with this resulting YFP signal were quantified with analyze particles (size, 0.5 μm^2^ to infinity; circularity, 0-1.00). The number of puncta were divided by the area of the YFP signal for final analysis. Significance of overlap with YFP was determined with a Wilcoxon–Mann–Whitney test comparing each probe to the no-probe control. Planned comparisons were also conducted across cell types.

The following probe primers were used: *Sst* (RP_090901_02_A05): forward, ACGCTACCGAAGCCGTC; reverse, TAATACGACTCACTATAGGGGGGGGCCAGGAGTTAAGGA; and *Cort* (RP_051101_02_C12): forward, AAACACCACAGAAGAGACCCTC; reverse, TAATACGACTCACTATAGGGGTTACTTGCACGAGGAGAAGGTT.

### Quantitative PCR

Three additional independent biological replicates of the WCH, TRAP, SNF, and ST were collected from each mouse line as described above and reverse transcribed using Quanta qScript Reverse Transcriptase (catalog #84002, QuantaBio). Three technical replicates of each of these samples and the five samples that were used in RNA-seq were quantified with Power UP iTaq Universal Sybr green (catalog #1725120, BIO-RAD) on a QuantStudio 6 Flex (Applied Biosystems) in a 10 µl volume with amplicons of <200 nt. β-Actin was used as an endogenous control. Statistical testing was determined by ANOVA with 5 df in R statistical software. Primer sequences from PrimerBank (X. Wang et al., 2012) were as follows: *Cort* [PrimerBank ID (PBID), 6680984a1]: forward, GAGCGGCCTTCTGACTTTCC; reverse, GGGCTTTTTATCCAGGTGTGG; *Sst* (PBID, 6678035a1): forward, ACCGGGAAACAGGAACTGG; reverse, TTGCTGGGTTCGAGTTGGC; and *Shank3* (PBID, 255918226c1): forward, CCGGACCTGCAACAAACGA; reverse, GCGCGTCTTGAAGGCTATGAT.

## Results

We aimed to investigate the local translation profiles of forebrain GABAergic neurons and pyramidal neurons of the cortex, two functionally and morphologically distinct neuronal types, using SynapTRAP. To target these neurons, we bred Cre-dependent TRAP reporter mice (Zhou et al., 2013) to the following two well characterized Cre lines: Tg(RBP4-cre)^KL100Gsat/Mmcd^, targeting layer 5 pyramidal cells (Beltramo et al., 2013); and Slc32a1^tm2(cre)Lowl^/J, targeting any GABAergic neurons, as defined by their expression of VGAT (Vong et al., 2011). Progeny are referred to as RBP4-TRAP and VGAT-TRAP. Immunofluorescence of the GFP-tagged ribosomal proteins shows robust expression in layer 5 pyramidal neurons for RBP4-TRAP mice (Fig. 1*A*,*B*) and a regularly distributed pattern in all layers, consistent with interneurons, for VGAT-TRAP mice (Fig. 1*C*,*D*). At higher magnification, GFP-tagged ribosomal proteins were also observed in the neurites of each line (Fig. 1*C*,*F*), indicating the potential to harvest ribosome-bound transcripts from this compartment. Therefore, from each line we collected five forebrain replicates for analysis of local translation. From each biological replicate, we harvested the following four samples: the total RNA from the region (WCH), RNA enriched from all ribosomes in neuron cell type of interest (TRAP), RNA from forebrain synaptoneurosomal fractions (SNF), and ribosome-bound RNA enriched from the neuron cell type of interest from the SNF (SynapTRAP:ST), as described previously (Ouwenga et al., 2017).

First, we verified that the harvest of mRNA by TRAP generated the expected enrichment characteristic of each cell type. TapeStation analysis revealed that TRAP and ST samples all showed the expected robust capture of both 18S and 28S ribosomal RNAs, indicating that the GFP-Rpl10a fusion protein was being incorporated into the large subunit in all fractions, and that these subunits were being recruited to 18S bound mRNA (data not shown), consistent with prior studies using this method on other cells (Ouwenga et al., 2017; Sakers et al., 2017). We then sequenced the transcripts from each sample using RNA-seq. Comparing standard TRAP to WCH RNA, each sample was well depleted for markers of glial cells (purple) and enriched for genes known to be expressed in cortical interneurons (blue) or projection neurons (red), respectively, confirming the enrichment of ribosome-bound RNA from each cell type (Fig. 2*A*,*B*, Fig. 2-1, Fig. 2-2). Further, directly comparing VGAT-TRAP to RBP4-TRAP mice identified thousands of transcripts (Fig. 2-3) enriched uniquely in the lines, as indicated by CSEA, a tool that compares the current gene lists to empirically defined lists of genes with “marker-like” expression in previously analyzed cell types at a variety of thresholds (Xu et al., 2014). In this analysis, the transcripts from RBP4-TRAP mice overlapped with markers derived from previous profiles of deep-layer projection neurons (Fig. 2*C*), with modest signaling from the Pnoc^+^ line, which labels a mix of projection and interneurons (Doyle et al., 2008). Likewise, the VGAT-TRAP profiles overlapped with prior profiles of Pnoc and cortistatin TRAP mice from cortex (Fig. 2*D*), lines confirmed to be expressed in interneurons (Nakajima, 2012). This overlap was driven by known markers of interneurons such as *Sst*, *Cort*, *Dlx1/2*, and *Htr3a*, as well as markers of projection neurons, such as *Fezf2* and *Slc17a7*, each of which showed robust enrichment in the appropriate cell type, particularly when they were directly compared (Fig. 2*E*). It was also clear from CSEA that the rapid forebrain dissection used for synaptoneurosome preparation here (Westmark et al., 2011; Ouwenga et al., 2017) likely included some dorsal striatum, as the VGAT-TRAP samples were also enriched in markers of GABAergic striatal Drd1 and Drd2^+^ medium spiny neurons, such as *Tac1*, *Adora2a*, *Drd1*, and *Drd2* (yellow), as well as markers of the subpopulation of Drd2 MSNs that are striatal cholinergic interneurons (*Chat*, *Slc18a3*), which are shared with other cholinergic populations (data not shown). Thus, we were confident we had enriched for ribosome-bound RNA from contrasting neuronal cell types.

We also verified that the fractionation for neurite RNAs enriched for individual transcripts previously identified as translated in neurites in neurons from other brain regions. Consistent with prior studies and our recent profiling of a pan-neuronal line in forebrain, we identified *Psd95* (*Dlg4*) as enriched in the SNF (Westmark et al., 2011) and *Shank3* as enriched in the ST (Cajigas et al., 2012) within all replicates of both the VGAT and RBP4-TRAP lines. This confirms that these genes previously defined as locally translated in neurons from other brain regions are in the current cell types and provides evidence that the method will be effective in these cell types.

### Characterization of local and somatic translation profiles of the neuronal cell types

To identify candidate transcripts showing robust local translation in each cell type, we applied an analysis to select for transcripts that are both enriched in the SNF and bound to ribosomes in the cell type of interest (Fig. 3), per our published strategy (Ouwenga et al., 2017). This intersectional analysis selects for transcripts that were both enriched in the SNF compared with the WCH (showing enrichment in neurites) and enriched in the ST compared with the SNF (on ribosomes from the targeted cell type). From this, RBP4-TRAP neurons had 247 local translation candidates (Fig. 3, Fig. 3-1) and VGAT-TRAP neurons had 480 candidates (Fig. 3*A*, Fig. 3-2). For contrasting controls, we generated a list of candidates for transcripts whose translation is predicted to be sequestered to the somatic, non-neurite, region of the neuron. These “somatic localization” candidates are defined as the intersection of transcripts enriched in the TRAP sample compared with the WCH sample and depleted in the SNF sample compared with the WCH sample (Fig. 3*B*, Fig. 3-3, Fig. 3-4). Note here that we are using the word “somatic” as a shorthand for “depleted in the SNF” as true physical dissection of somas from neurites in densely intermingled regions such as the cortex would not be feasible (see also Discussion).

Comparing the two local translation profiles shows that the local transcripts are similar across two neuronal types and that both lists include transcripts known to be translated locally in the hippocampus, such as *Camk2a* and *Shank3*, and a gene ontology (GO) pathway analysis reveals fairly similar pathways showing enrichment of these transcripts (Fig. 4*A*,*B*). Overall, the two cell type lists overlap by 36% of transcripts (Fisher’s exact test, *p* < 0.0001), and GO analysis of these genes highlights cell projection (*p* = 2.96E-10) and cell junction proteins (*p* = 2.3E-10) as common themes (Fig. 4*C*, Fig. 4-1, Fig. 4-2, Fig. 4-3). This suggests that both neuronal cell types use some similar pathways for general synaptic maintenance and function, and that there is a baseline of the local translatome that is shared across neurons.

10.1523/ENEURO.0320-18.2018.f4-4 Figure 4-4 GO analysis results of RBP4 TRAP vs WCH enriched transcripts with a significance cutoff of *p* = 5E-8. Download Figure 4-4, XLS file.

**Figure 5.**
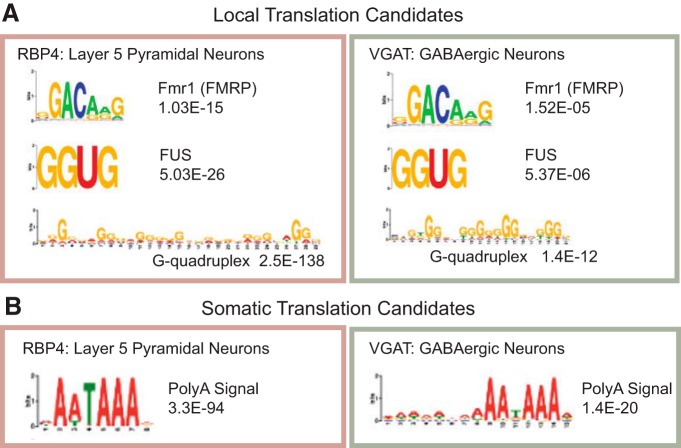
Protein binding motifs are enriched in local and somatic candidate lists. ***A***, A MEME analysis reveals that Fmr1, FUS, and G-quadruplex binding motifs are enriched in local translation candidates of both cell types compared with somatic translation candidates as control. ***B***, A MEME analysis reveals that the Poly A signal sequence is enriched in somatic translation candidates of both cell types compared with local translation candidates as a control.

10.1523/ENEURO.0320-18.2018.f4-5 Figure 4-5 GO analysis results of VGAT TRAP vs WCH enriched transcripts with a significance cutoff of *p* = 5E-8. Download Figure 4-5, XLS file.

### Shared features of local candidate transcript sequence across the neuronal cell types

Examining the sequences of the transcripts identified common features for local translation candidates across both pyramidal neurons and GABAergic neurons. Similar to prior results in a pan-neuronal line (Ouwenga et al., 2017), the local translation candidates had 3´ UTRs that were both longer and had higher GC content when compared with the somatic candidates. Longer 3´ UTRs may allow for more binding sites of proteins and miRNAs for the regulation of translation and localization, while higher GC content may influence the stability of these transcripts as they are shuttled to neurites (Wu and Brewer, 2012).

As these regions could contain regulatory sequences that act as binding sites for RNABPs mediating localization or translational control, the 3´ UTRs were analyzed for enriched novel motifs using MEME (McLeay and Bailey, 2010), which identifies enriched sequences in the local translation candidates of each cell type versus their own somatic translation candidates. Similar to previous findings, we again find a motif that highly resembles a G-quadruplex in local candidates (Fig. 5*A*), which is a sequence known to interact with the RNABP fragile X mental retardation protein (FMRP). There is also a poly-A binding motif enriched in somatic candidates (Fig. 5*B*).

Additionally, the 3´ UTRs of the local candidate were analyzed using AME, a tool to identify binding motifs for known RNA binding proteins in a candidate list. Both cell types were enriched for another FMRP binding motif (GGACAAG: VGAT, *p* = 1.5E-05; RBP4, *p* = 1E-15) as well as a binding site for FUS RNA binding protein (GGUG: VGAT, *p* = 5.3E-06; RBP4, *p* = 5E-26; Fig. 5*A*), a gene clearly implicated in amyotrophic lateral sclerosis (ALS; Deng et al., 2014). Although FUS has mostly been associated with the binding of introns during the transcription of long, neuron-expressed genes in the nucleus, 8–10% of FUS binding occurs on 3´ UTRs (Lagier-Tourenne et al., 2012), suggesting that it may also have roles in post-transcriptional regulation. Indeed, it has been shown to be involved in the post-transcriptional regulation of the mRNA of an AMPA receptor subunit (GluA1; Udagawa et al., 2015). Enrichment here suggests that it may have a similar role for dozens of other proteins as well. Overall, the similarities in binding motifs enriched in the 3´ UTRs across the cell types suggests that multiple neuronal cell types share similar pathways in mRNA regulation.

### Identification of differential localized translation between the distinct cell types

**Figure 6.**
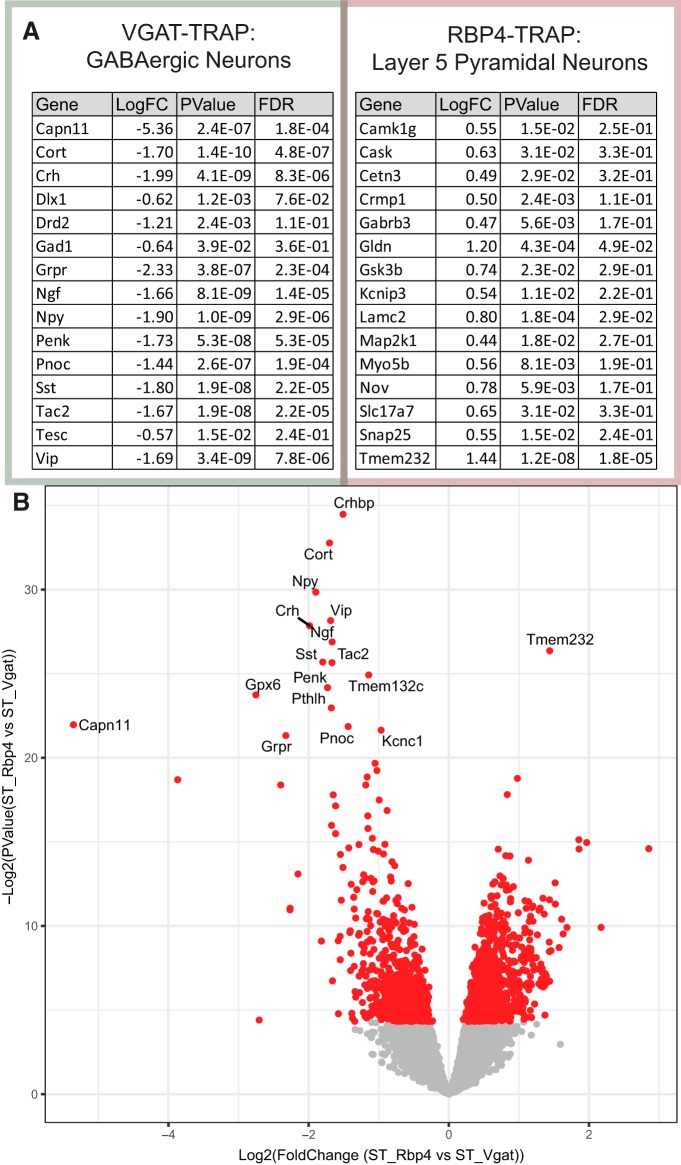
Direct comparison reveals quantitative differences in SynapTRAP between RBP4 and VGAT neurons. ***A***, Table of 15 representative neurons with quantitative differences between the local translatome of the cell types as identified by a directed differential expression of ST samples. ***B***, Volcano plot of differential expression results from the direct comparison of ST between cell types. Genes with significant differences between cell types (*p* < 0.05) are shown in red. Figure 6-1: Table of Cell Type Specific Local Translation Candidates in Pyramidal Neurons (RBP4-TRAP). Figure 6-2: Table of Cell Type Specific Local Translation Candidates in GABAergic Neurons (Vgat-TRAP).

**Figure 7.**
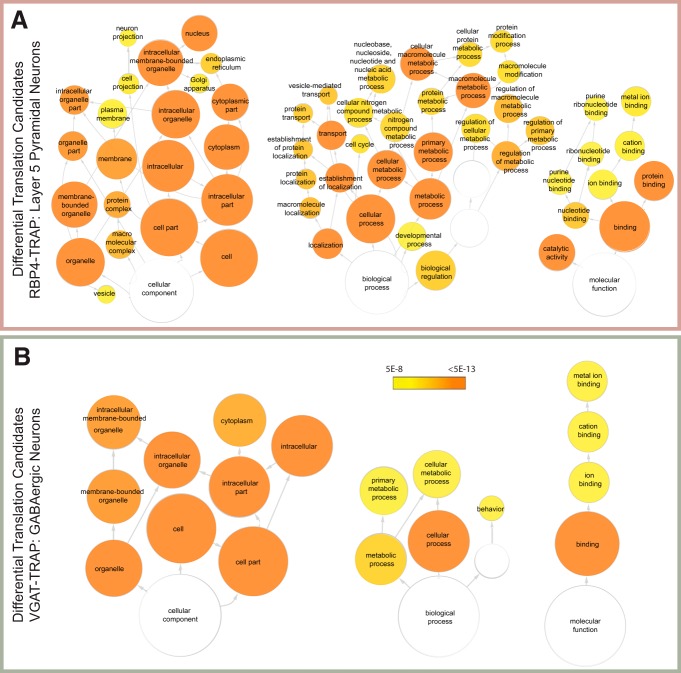
GO analysis of quantitatively enriched SynapTRAP candidates. ***A***, A gene ontologies analysis of local translation candidates quantitatively enriched on RBP4-ST samples over VGAT-ST. ***B***, A gene ontologies analysis of local translation candidates quantitatively enriched on VGAT-ST samples over RBP4-ST. Figure 7-1: GO analysis results of RBP4-TRAP Differential local translation candidates Significance cutoff *p* = 5E-8. Figure 7-2: GO analysis results of VGAT-TRAP Differential local translation candidates Significance cutoff *p* = 5E-8.

Although many transcripts are clearly shared, we next designed an analysis to specifically identify the quantitative differences in local transcripts between these types of neurons by direct statistical testing for differential expression between the ST samples. Differential analysis between the ST samples of the pyramidal and GABAergic neurons showed that these included markers of each cell type (Fig. 6*A*), but also identified hundreds of quantitative differences in the profiles of ribosome-bound transcripts in the SNF for each (Fig. 6*B*). We used gene ontologies to gain a systematic view of the data. This analysis of the pyramidal neuron candidates identified a variety of enriched terms, including neuronal projection (Fig. 7*A*; *p* = 6.4E-9), highlighting the need for that maintenance of long neurites. Gene ontology analysis of the GABAergic neuron differential translation candidates identified an enrichment of behavior (Fig. 7*B*; *p* = 2E-8), highlighting neuropeptides and dopamine receptors as potential locally translated proteins that modify higher-order actions.

10.1523/ENEURO.0320-18.2018.f6-1 Figure 6-1 Table of cell type-specific local translation candidates in pyramidal neurons (RBP4-TRAP). Table of transcripts quantitatively enriched in pyramidal neurons (RBP4-SynapTRAP) compared with GABAergic neurons. Table containing RBP4-TRAP-enriched transcripts from a direct EdgeR comparison of ST fractions between cell types. Column names are as in Figure 2-1. Download Figure 6-1, XLS file.

10.1523/ENEURO.0320-18.2018.f6-2 Figure 6-2 Table of cell type-specific local translation candidates in GABAergic neurons (VGAT-TRAP). Table of transcripts quantitatively enriched in GABAergic neurons (VGAT-SynapTRAP) compared with pyramidal neurons. Table containing VGAT-TRAP-enriched transcripts from a direct EdgeR comparison of ST fractions between cell types. Column names are as in Figure 2-1. Download Figure 6-2, XLS file.

10.1523/ENEURO.0320-18.2018.f7-1 Figure 7-1 GO analysis results of RBP4-TRAP differential local translation candidates with significance cutoff of *p* = 5E-8. Full table of GO analysis results of RBP4-TRAP-enriched candidates with significance cutoff of *p* = 5E-8. Column headers are as in Figure 4-1. Download Figure 7-1, XLS file.

10.1523/ENEURO.0320-18.2018.f7-2 Figure 7-2 GO analysis results of VGAT-TRAP differential local translation candidates with significance cutoff of *p* = 5E-8. Full table of GO analysis results of VGAT-TRAP-enriched candidates with significance cutoff of *p* = 5E-8. Column headers are as in Figure 4-1. Download Figure 7-2, XLS file.

### Features of candidate transcript sequences that differ across neuronal cell type

**Figure 8.**
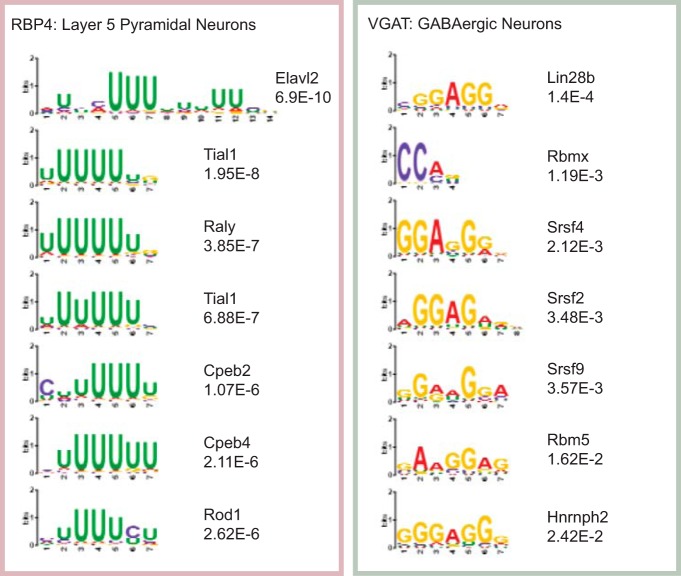
Distinct enrichment of RNA binding motifs in transcripts with quantitative SynapTRAP differences between cell types. A MEME analysis identifies known RNA binding protein motifs enriched in the 3´ UTR of transcripts with quantitative differences in ST between RBP4 and VGAT. *p* values were Bonferroni corrected .

If post-transcriptional regulation does indeed play a role in local translation, then sequences enriched in each neuronal type might have distinctive *cis* regulatory motifs. We hypothesized that these transcripts with differences between the cell types may be guided to neurites through a cell-specific RNA binding motif. We tested this hypothesis by scanning the 3´ UTRs of the differentially localized transcripts for known binding motifs using AME (Fig. 8). This additional level of regulation of transcripts further divides different neuronal types from each other and provides support for a role of post-transcriptional regulation in defining the local translatome of each distinct cell type. However, due to the similarity of motifs bound by distinct RNABPs, it is difficult to predict from sequence alone which proteins might actually be binding these motifs in each cell type.

### The local translatome is defined by both transcriptional and post-transcriptional regulation

**Figure 9.**
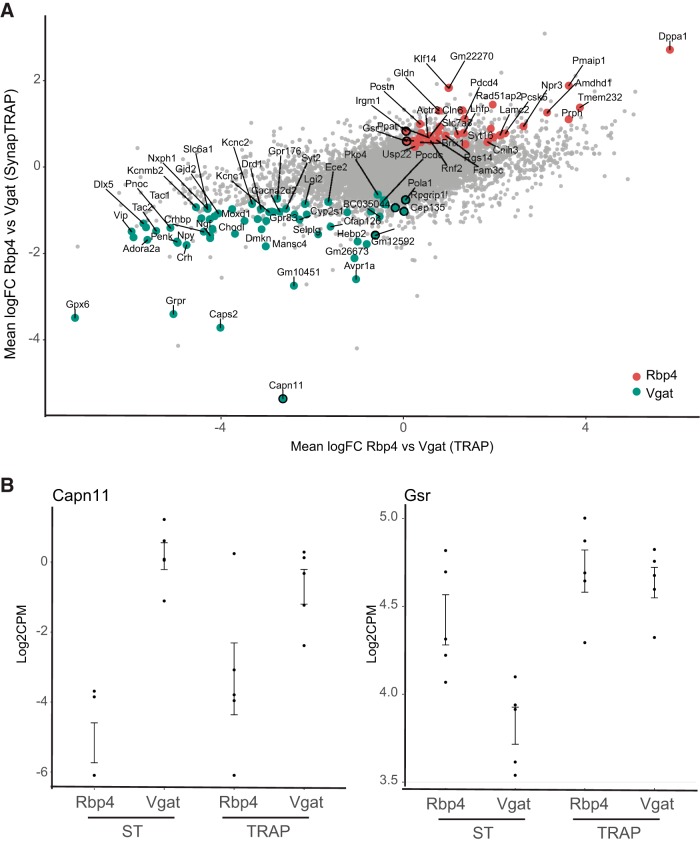
Quantitative regulation of SynapTRAP RNAs occurs by both differential baseline TRAP expression and differential localization. ***A***, Differential expression analysis reveals a subset of genes significantly differentially expressed between RBP4 and VGAT SynapTRAP samples may be mediated by differential neurite localization rather than differential TRAP expression. Log2-transformed fold-change (logFC) between SynapTRAP samples is shown on the *y*-axis, and logFC between TRAP samples is shown on the *x*-axis. Deviation from perfect correlation indicates additional post-transcriptional regulation. Transcripts with significant evidence for such regulation are shown in red (RBP4 upregulated) and blue (VGAT upregulated), respectively. ***B***, Representative examples of transcripts showing robust post-transcriptional regulation. Both *Capn11* and *Gsr* show differences in their SynapTRAP RNA-seq expression that are not reflected in TRAP RNA-seq. Figure 9-1: Table containing differential expression statistics for all direct comparisons between the two neuron types.

Cell-specific differences in localized translation may be different for two reasons. First, the transcript may not be transcribed in the particular neuronal type. For example, we detect *Sst* and *Cort* transcripts here as enriched in VGAT SynapTRAP. While *Sst* and *Cort* are both highly abundant in subsets of cortical interneurons (Schindler et al., 1996; de Lecea et al., 1997), they are largely not transcribed in pyramidal cells, thus making it impossible for the transcript to reach the neurites. The second reason would be cell-specific post-transcriptional regulation such as transcript-specific differences in RNA localization. As hinted at in the differences of the 3´ UTRs of local candidates, we suspected that cell-specific machinery may be driving the localization of a subset of candidates to neurites. Indeed, plotting the ratios of TRAP versus TRAP for each cell type, compared with ST versus ST, reveals a number of transcripts that deviate in their ST from what TRAP would have predicted (Fig. 9*A*). Gsr, for example, is expressed in both cell types by TRAP; however, it is not found in the neurites of GABAergic neurons by ST (Fig. 9*B*). However, the largely shared GO processes (Fig. 4) and motifs (Fig. 5) suggested that baseline differences might outweigh localization-mediated differences.

We therefore systematically tested this hypothesis. We identified transcripts undergoing cell type-specific spatial regulation using a statistical approach to distinguish baseline transcript levels from differential localization using the RNA-seq data; we sought both to determine the extent to which differences in local translation, as assessed by ST, were already present in the corresponding TRAP sample or were independently arising due to differential localization to ST across cell types. We found that the majority of the differences between cell types are likely attributable to baseline differences: of the 1631 transcripts identified as differentially expressed between ST fractions of the two neuronal cell types at *p* < 0.05, 1396 appeared to be due to expression differences also apparent in TRAP. However, the remaining 235 transcripts (14.4%) appeared to be mediated by differences in RNA localization or localization to ribosomes in ST between cell types (Fig. 9*A*, 9-1). Thus, focusing on the 99 top differentially expressed genes between ST fractions, 7 still showed differential localization between cell types, notably, Capn11 (Fig. 9*B*, 9-1). Thus, while baseline transcript differences are preponderant, there is also a role for cell type-specific translational regulation in determining the localized translatome.

### mRNAs for neuropeptide neurotransmitters are localized to neurites

**Figure 10.**
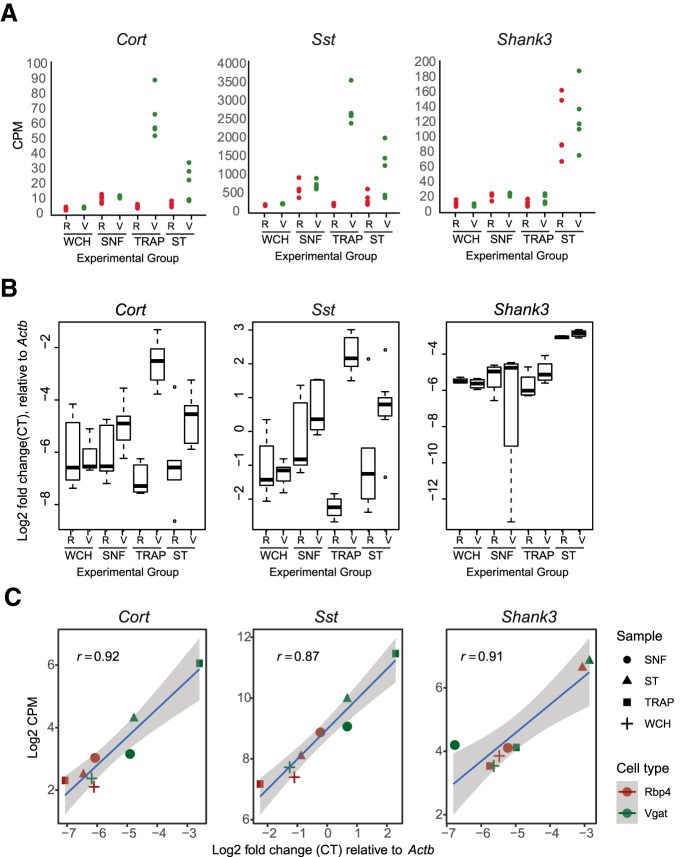
qPCR validation of neuropeptide RNA in neurites. ***A***, RNA-seq-measured expression level, normalized by counts per million, shows enrichment of neuropeptides *Cort* and *Sst* in both the TRAP of ST specifically of VGAT neurons. *Shank3*, a well characterized locally translated gene found enriched in both RBP4 and VGAT ST, is included as a control. ***B***, qPCR results of four additional biological replicates for the neuropeptides *Cort* and *Sst* confirm the enrichment of messages for both peptides in both the TRAP and ST fractions from VGAT neurons. R, RBP4; V, VGAT. qPCR results are normalized to *Actb* mRNA using the dCT method. ***C***, Scatterplots of Log2CPM of RNAseq compared to qPCR in replicate samples confirms reproducibility of the findings.

Finally, previous models of neuropeptide precursor protein biosynthesis have described their translation as occurring only in the soma. The enrichment of these mRNAs in the ST fraction suggests a secondary location for the synthesis of these proteins (Fig. 10*A*). To validate the localization of mRNA for neuropeptides in the neurites of inhibitory neurons, additional biological replicates underwent qPCR for *Sst* and *Cort* mRNAs. As a positive control, *Shank3* showed the expected local translation candidate from both cell types and was expressed at relatively similar levels in the both ST samples. All candidates replicated the RNA-seq results (Fig. 10*B*).

**Figure 11.**
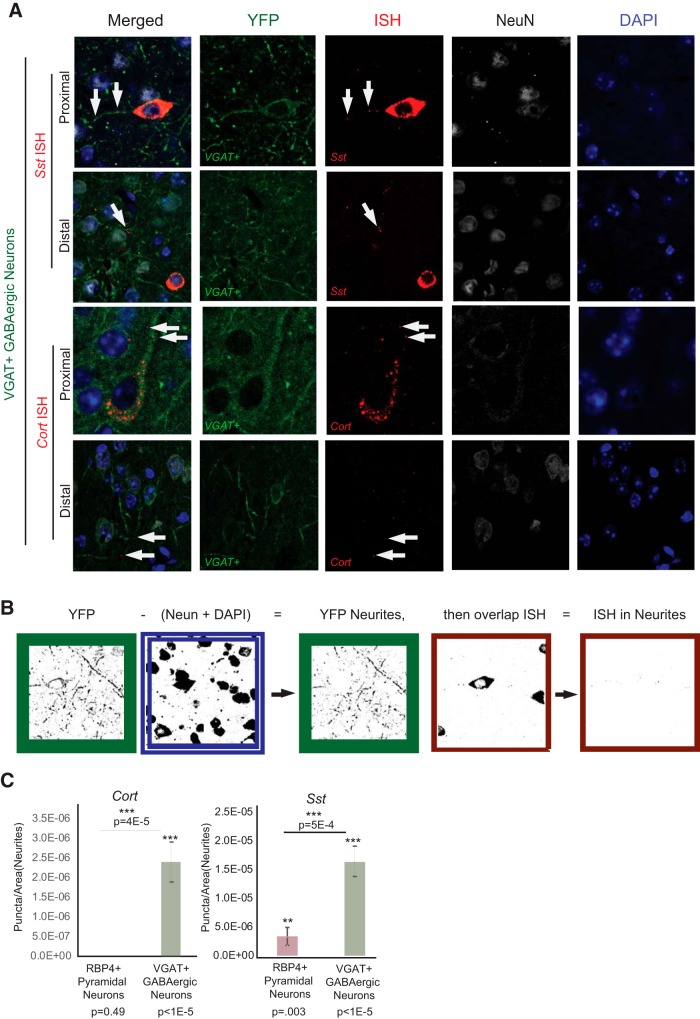
*In situ* hybridization validation of neuropeptide mRNA in neurites. ***A***, *In situ* hybridization shows RNA localization for *Sst* and *Cort* messages with immunohistochemistry of Cre-dependent membrane-bound YFP-channel fusion in each cell type. DAPI is used to label nuclei, and NeuN to define the nuclear compartment and perinuclear cytoplasm of all cortical neurons. White arrows indicate examples of *Cort* and *Sst* ISH puncta overlapping with both proximal and distal neurites in subsets of VGAT neurons. ***B***, Illustration of the method used to quantify the ISH signal in the neurites of each cell type. The cell-specific Cre-driven YFP signal (green border) was masked with NeuN and DAPI (blue and white border) to remove any signal in nuclear and perinuclear compartments. The remaining ISH puncta overlapping with GAP (red borders) were quantified. ***C***, Quantification of overlapping ISH puncta with cell-specific labeling of neurites reveals that *Cort* and *Sst* are significantly enriched in neurites of VGAT neurons. Each probe and no probe control, *n* = 13, Mann–Whitney test. **P < 0.01, ***P < 0.001.

The neuropeptide mRNAs were also confirmed to colocalize with GABAergic neurites by ISH (Fig. 11). To visualize individual neurites in a cell-specific manner, a CRE-dependent YFP [AAV5-EF1a-DIO-hChR2(H134R)-EYFP] was injected into the VGAT-CRE and RBP4-CRE mouse lines. This produced cell-specific labeling of these neurons. Labeling of NeuN and DAPI was used to mask nuclear and perinuclear soma, enabling the quantification of ISH puncta overlapping with the neurites of each cell type. Quantification revealed a measurable and consistent presence of these messages in the neurites of GABAergic cortical neurons (Fig. 11).

## Discussion

Local translation is instrumental in many neuronal functions from the formation of neurites to the regulation of synapses in response to activity (Kislauskis et al., 1993; Sutton and Schuman, 2005; Lin and Holt, 2007). We contrasted cortical layer 5 projection neurons to GABAergic neurons to investigate the extent to which local translation is distinctly regulated and found evidence for multiple mechanisms defining the differences between these types of cells. We found, similar to studies that look at whole-cell translation by TRAP, that which transcripts show ribosome occupancy in neurites changes across neurons as well. While there is substantial overlap between these local translation candidates of the two cell types, the majority are neuron type specific. Understandably, with the contrasting functions (i.e., inhibitory vs excitatory) of these two neuron types, one might expect key differences among the pool of mRNAs being translated in the neurites at each cell.

There are two main ways in which distinct cell types could regulate these differences in localized translation. First, differences could simply reflect baseline differences between the cell types, such as those that would be driven by differences in transcription or splicing in the nucleus. Second, there could be a level of post-transcriptional regulation specifically impacting local translation (i.e., altered mRNA localization or ribosome occupancy in neurites). We found that the majority of differences between cell types when analyzing mRNAs bound to ribosomes in the SNF fraction was already apparent in the standard TRAP sample. Thus, it seems likely that most of the differences are already apparent in the cell body and is thus transcriptional. However, there is still a clear role for cell type-specific regulation of localization to SFN ribosomes in a subset of transcripts. Approximately 14% of transcripts showed deviations in ST that were not predicted from the TRAP data, indicating that post-transcriptional mechanisms were altering ribosome occupancy in neurites in a cell type-specific manner. This was exemplified by transcripts like *Gsr* and *Capn11*, which were expressed by TRAP in both cell populations but were predominantly ribosome-bound in neurites by only one of the two.

An unexpected finding from our analysis was the possible cell-specific local translation of two neuropeptide transmitters, cortistatin and somatostatin, in the GABAergic neurons. These are examples of SynapTRAP differences reflecting transcriptional difference between the two cell types in that they are transcripts that are expressed in GABAergic neurons but not in pyramidal neurons. While the processed neuropeptides of these mRNAs have been a marker of inhibitory neurons in the cortex (Close et al., 2017), they have never before been characterized as potentially locally translated in interneurons. These two neuropeptides have similar structure, act as inhibitory neuropeptides, and bind many of the same G-protein-coupled receptors, although they have distinct physiologic functions (Spier and de Lecea, 2000; de Lecea and Castaño, 2006). Somatostatin was identified for its ability to inhibit growth hormone release (Brazeau et al., 1973), and cortistatin for its induction of slow waves in the cerebral cortex related to sleep (de Lecea et al., 1996).

Previous literature describes neuropeptide precursor mRNA being translated in the endoplasmic reticulum (ER) of the soma and with the immature precursor protein transported down neurites in vesicles where they are then processed into mature neuropeptides (Goodman et al., 1983; Hökfelt et al., 2000; Russo, 2017). This model was developed through several methods including axonal injury and disruption of microtubular transport. Specifically, on axonal injury, neuropeptides build up proximal to the point of injury. Furthermore, when the axon was injured in two locations, an additional, but smaller, buildup of neuropeptides occurred between the two injury sites (Gilbert et al., 1980). Although not discussed by the authors at the time, these data confirm that local synthesis of neuropeptides in neurites is possible, though whether this was mediated by mRNA translation or peptide precursor processing is not clear from this experiment alone. However, as ER and Golgi structures extend into neurites (Tsukita and Ishikawa, 1976; Henkart et al., 1978; de Juan-Sanz et al., 2017), the components needed for neuropeptide synthesis from mRNA to neuropeptide are present locally. Additionally, classic studies of neurons treated with colchicine, a microtubular disruptor, were interpreted as disrupting peptide localization to neurites because they disrupted precursor vesicle transport; however, this treatment could also inhibit the transport of mRNA and ER dynamics, preventing the trafficking of mRNAs to the neurites as well as the vesicles of precursor protein. Thus, the data supporting the old model are not inconsistent with local translation of neuropeptide precursor mRNA occurring in neurites in addition to the soma.

Indeed, several studies have speculated about the presence of neuropeptide mRNA in neurites; however, they lacked a method for cell type-specific labeling and finer resolution microscopy to confirm it (Lehmann et al., 1990; Levy et al., 1990; Mohr et al., 1990). The cell-specific expression and localized enrichment of *Cort* and *Sst* levels were confirmed here through qPCR (Fig. 10*A*) in independent samples. In addition, fine-resolution fluorescent confocal microscopy confirmed cell type specificity and localization by ISH puncta of both neuropeptide precursor mRNAs overlapping with cell-specific labeling of neurites from the VGAT-CRE mouse line compared with no probe and the RBP4-CRE lines (Fig. 11*C*). The additional pathway of local production of these neuropeptides in neurites would allow for greater temporal and spatial control of neuropeptide release. Unlike the fast neurotransmitter glutamate, neuropeptides are degraded rather than recycled after release, requiring new protein to be generated for continued signaling. Localized translation of these peptides could allow for a more rapid replenishment compared with shuttling precursor proteins from the soma.

Our study did have at least three limitations. First, while these results demonstrate that there must be a diversity in local translation across cell types, samples isolated from the VGAT-TRAP line (GABAergic neurons) plausibly include multiple more precisely definable cell types of GABAergic neurons of the cortex and striatum (Nakajima, 2012). Regardless, even without focusing on more precise subtypes of GABAergic neurons, there are robust differences with layer 5 pyramidal neurons . These new findings extend previous knowledge of local translation gathered from cell types that are laminarly organized, such as the hippocampus (Zhong et al., 2006; Taylor et al., 2009; Cajigas et al., 2012), and suggest that there are both consistent sets of genes that might represent a fairly “core local translatome” as well as neuron type-specific differences. Further investigation of local translation in a greater number of cell types may reveal more regulatory pathways of this localized phenomenon. It might be of particular interest to focus on neuron types with highly unique polarized processes, such as the clawed dendrites of cerebellar granule cells, or the magnificent specialization that is the calyx of Held in the auditory colliculus. In addition, profiling more defined cell types may reduce the variation and allow for a deeper analysis of the mechanisms of those transcripts that do show post-transcriptional regulation. With the vast array of TRAP and Cre mouse lines available, the ability to investigate localized translation in large a survey of cell types is possible.

Likewise, differential splicing is one method that cells may use to localize some isoforms to neurites (Taliaferro et al., 2016). Due to the use of 3´ priming in library preparation, this dataset is not well suited for the discovery of differentially localized isoforms as it has a heavy 3´ bias in sequencing reads. In addition, our analysis of motifs that might mediate RNA localization was limited by our simplifying assumption focusing on the longest transcript from each gene for motif discovery, and the validity of our motif results are contingent on this assumption being a reasonable one. Alternative library preparation methods might provide more insight into isoform localization and enable more thorough analysis of key regulatory motifs. However, cell-specific splicing of transcripts would be an additional level of regulation to alter local translation across cell types that could factor into the specialized functions of each cell.

Finally, we also cannot determine which messages in the SNF might be coming from axons, and which from dendrites. A prior study (Shigeoka et al., 2016) was able to elegantly profile axon-specific ribosome-bound mRNA by separately conducting TRAP on regions containing either cell bodies or axons using neurons with long-range projections. Similarly, our term somatic is operationalized as simply being “non-SNF.” It may indeed still contain some neurite fragments, so it is not as pure a measure of a somatic fraction as Shigeoka et al. (2016) were able to achieve. However, for cells such as cortical GABAergic interneurons, where axons only project locally, we would not be able to use such a physical separation approach. Furthermore, we have assessed only ribosome occupancy (by TRAP and ST) rather than a direct measure of active protein translation. With current methods available *in vivo*, we cannot strictly determine the difference between actively translating transcripts versus transcripts that might be stalled but still bound by ribosomes in neurites.

Nonetheless, the current findings indicate an array of interesting future avenues of investigation. Notably, the developmental differences in cell-specific local translation could be explored. In the current analysis, the candidates for local translation were identified in mice at 21 d postbirth. At that age, the cortical neurons have finished their developmental migrations. Given the two contrasting migratory directions of the two cell types (Molnár et al., 2006), it is possible that additional cell-specific candidates in neurites would be identified at earlier time points that aid in cell polarity and motility during development. Likewise, it would be of interest to determine whether there are cell type-specific specializations in the localized translation in response to activity in the mature nervous system. This could be important for any role in local translation in changes in synaptic strength, learning, and memory. Further, if the method could be successfully adapted to applications in the spinal cord, it would be of interest to assess how the neurite translatome profiled might be altered during the neurodegenerative disease ALS. Indeed, it has been show that each of the major cell types (astrocytes, oligodendrocytes, and neurons) has a distinct translational response in models of ALS (Sun et al., 2015); however, it is not clear to what extent this might be occurring in peripheral processes compared with the whole cell.

Finally, this article describes local translation in normal early postnatal neurons. While many neurologic diseases, such as fragile X syndrome, which is caused by a lack of FMRP, have been associated with altered local translation (Kelleher and Bear, 2008; Meyer-Luehmann et al., 2009), it is unclear whether disease causes the same perturbation in all neurons or whether a certain cell type contributes more heavily to the phenotypes. With FMRP binding motifs enriched in the 3´ UTR of the local translation candidates of both cell types, disruption of this master regulator would have a broad effect on local translation, but it is unclear how this and other diseases alter local translation on a cell-specific level across the CNS. Thus, determining how mutations modeling RNABP disease impact local translation in a cell type-specific manner would also be of substantial future interest.

10.1523/ENEURO.0320-18.2018.f9-1 Figure 9-1 Table containing differential expression statistics for all direct comparisons between the two neuron types. Log-fold change (logFC), *p* values, and adjusted *p* values (FDR) for each of three EdgeR comparisons. Statistical comparisons were performed between RBP4 and VGAT SynapTRAP samples (PValue.ST_RBP4vVGAT), as well as between RBP4 and VGAT WCH samples (PreIP_RvV), as well as an interaction term, which tests the extent to which cell type differences in the ST fraction are not found in TRAP fractions, indicating substantial post-transcriptional regulation. Download Figure 9-1, XLS file.

## Synthesis

Reviewing Editor: Katalin Toth, Universite Laval Decisions are customarily a result of the Reviewing Editor and the peer reviewers coming together and discussing their recommendations until a consensus is reached. When revisions are invited, a fact-based synthesis statement explaining their decision and outlining what is needed to prepare a revision will be listed below. The following reviewer(s) agreed to reveal their identity: Gabriella Viero, Charles Plessy.

While both reviewers found the presented data valuable, the manuscript had two major flaws.

1. Reviewers were not able to see the figures properly due to the poor resolution.

2. The manuscript has several overstatements that need to be either removed or modified in light of the reviewers' comments.

Rev. #1.

I read with very much interest the manuscript. The working hypothesis is worth exploring and up to date.

Unfortunately, the results obtained are not always clearly presented and explained, the conclusions are not supported by the data as they are presented in the present form of the manuscript. There a number of major problems, some of them are listed below:

1. Figures. All Figures are presented at such low resolution that the reader cannot independently look at the results by himself. Major examples are Figure 2 panel E, where in the scatter plot the gene names cannot be read; Figure 4 and Figure 7 are possibly even worse than Figure 2E; same problem with Figure 9A and the labels in the plot in Figure 10 and all Figure 11. All fluorescence data are not clear. Overall this was very upsetting. Moreover the captions of all figures are too short and not very well prepared, making it not possible to understand what is shown in figure. Moreover there are a number of problems in the citation order of the Figures in the manuscript (for the example panels Figure 5A and B are cited in reversed order, panels in Figure 6 are not commented in the results; Figure 9A is not even mentioned). I strongly encourage the authors to submit better figures and to revise captions and manuscript, otherwise the comprehension of the results is too poor.

2. There are a number of clear overstatements about the interpretations of some results, especially from the point of view of translation studies. Therefore, for the sake of clarity some statements should be removed, other toned down. Specifically:

a. Line 324: Ribosomes cannot be resolved using fluorescence microscopy. One ribosome has, roughly speaking, a diameter of 25nm. This is one order of magnitude below the resolution of the best confocal microscopy (see Abbe's law). This means that neither polysomes (around 200 nm mean value) nor ribosomes can be distinguished from a tagged ribosomal protein which is NOT incorporated into the ribosomal subunit and/or ribosomes. If the authors want to demonstrate their claim by microscopy techniques they should use super-resolution approaches such as STED or STOM. Moreover, if they want to demonstrate that their RPL protein is incorporated in ribosomes and polysomes, they should use sucrose gradient fractionation and western blotting. Therefore I strongly suggest to remove all sentences connected with “demonstration” that their cell model is incorporating the protein into ribosomes or to do the abovementioned experiments.

b. Line 337: the authors claimed that they “confirmed enrichment of ribosome bound mRNA from each cell type”. This conclusion was drawn citing Figure 2A and B, but there is not such demonstration. What the authors showed is the enrichment of mRNAs (which can be purified in any case form beads). Therefore, as explained in the point a. above, I think that the authors should tone down the “ribosome-bound”. Again, to demonstrate this, the authors should have performed at least a WB on their IP, showing that, compared to controls (i.e. IgG or to the input they used for transcriptome analyses) their IP samples were enriched in proteins associated to ribosomes and polysomes (i.e. RPLs, RPSs, eIFs, etc...).

c. Line 369-370, again I would suggest to tone down the “ribosome” bound concept.

d. Line 522 and 596: The authors discussed about “ribosome occupancy”. Actually this concept/parameter is used when performing ribosome profiling not TRAP or POL-Seq. Therefore this concept should be removed because not correctly used.

e. Line 596: The authors mentioned their technique as a “proxy for active translation”. Despite toned down in the following sentence, this is wrong and clearly an overstatement especially in neuro-tissues. There are a number of publications and it is well known that translation is often locally silenced in cells. Interestingly, major examples are ribosomes bound by FMRP, which is known to act as a translational repressor in brain (see Darnell et al., 2011). Therefore, this tone down is even more important I light of the fact that the authors showed an enrichment in motifs bound by FMRP, therefore one would rather suggest that they are purifying stalled ribosomes and not active ones! This is exactly the opposite of what the authors are claiming in this sentence.

f. Line 611: the authors conclude that “this paper describes local translation in normally developing neurons”. As it is, the sentence is somehow misleading because it seems that they characterized the translatome during development, that is not the case. I guess that simply stating in “control neurons” (or similar) would be clearer.

g. Line 504-5: the statement “found evidence” is somehow too strong given the observations made by the authors themselves in line 418, 387 and 507.

3. The authors often used the term “somatic” referring to their analysis. This concept is not discussed. The authors should mention and comment and cite the work by Shigeoka et al (Shigeoka T, Jung H, Jung J, Turner-Bridger B, Ohk J, Lin JQ, Amieux PS, Holt CE. Dynamic Axonal Translation in Developing and Mature Visual Circuits. Cell. 2016 Jun 30;166(1):181-92.) by the Holt's lab where they did actually use an anatomical separation of axons and cell bodies to apply TRAP and obtain “true” local translatomes. Therefore I strongly suggest the authors to tone down their “somatic” concept, at least citing and discussing the abovementioned work highlighting the limitation of their approach. In line with these observations, the use of “local translation” in line 528, 531-532 should be discussed better.

4. This very same observation is also true about the cell specificity. In fact, again, the authors did not mentioned the work by Cleveland's lab (Sun S, Sun Y, Ling SC, Ferraiuolo L, McAlonis-Downes M, Zou Y, Drenner K, Wang Y, Ditsworth D, Tokunaga S, Kopelevich A, Kaspar BK, Lagier-Tourenne C, Cleveland DW. Translational profiling identifies a cascade of damage initiated in motor neurons and spreading to glia in mutant SOD1-mediated ALS. Proc Natl Acad Sci U S A. 2015 Dec 15;112(50):E6993-7002.) which applied TRAP to track translatomes in motor neurons, oligodendrocytes and astrocytes. I think that, despite cell types are different as well the specific goals, this paper should be mentioned and discussed. In fact in this paper the authors conclude that “mRNAs purified from the three cell types of nontransgenic mice revealed [...] a clear distinction of mRNAs from motor neurons, astrocytes, oligodendrocytes, and whole spinal cord”, how do the authors relate this result to theirs?

5. In Figure 10 the authors wanted to validate the NGS data by qPCR. To do that they should do a scatterplot comparing the two and calculate the correlation. At present the qPCR data are shown as FC (no clear of what, i.e. not clear what was the reference) while the NGS data are shown as CPM. Thus, in the present form it seems that the authors chose different representation to shown the same “trend” by eyes. Since I am sure this was not the case, a scatterplot would be helpful to prove their statement in line 487.

6. In Material and Methods the authors mentioned EM and WB data but there are no such data.

7. It was not clear throughout the result sections and the figure legends what data are shown, i.e. if they are referring to the comparison between somatic/local (neurites) candidates or differential analysis between cell type specific candidates.

Minor comments:

1. In the title I guess that “is” should be “are

2. Not clear what do the authors mean in the title by “baseline cellular differences”

3. Line 25: “across neuronal cell types” is an overstatement because they characterize 2 cell types.

4. Line 30: I do not think “created” is a proper concept in a scientific report, so I suggest to replace with “synthesized”.

5. Line 48: as explained in major 3 and 4 other work should be cited. Since much work has been done also in other cell types that hippocampal neurons, I guess that the sentence in line 48 should be rephrased.

6. Line 87: it's not clear why the authors focused on layer V pyramidal and GABAergic neurons. A short explanation is actually provided, though as the authors mention in lines 54-60 many other types of neuros differs for their translational profiles, morphological features and functions.

7. Line 111 not clear what do the authors mean by “are reported in the MM section”, since this is already the MM section.

8. Line 114 not clear what does it mean were performed with both sexes in each column. What do the column refer to?

9. Line 125: SNF please define using capital letter afterwards

10. Line 152: not clear the use of “somatic”, should be stated that it's not a purification from the soma of neurons.

11. Line 152 the authors mentioned 3 trap/st samples but in line 120 they said 5 replicas.

12. Line 159: MDS not defined

13. Line 171: not clear “differential” what does refer to

14. Line 178 cellular fractionation was never mentioned before, so I suggest to change with SNF that was defined before.

15. Line 191: too specific. I would remove, modifying the sentences before and after.

16. Lines 197-299: there should be also a formula for the TRAP contrast

17. line 198: it should be ST = ... for consistency with the formula in line 197

18. Line 214 “analysis for”, something missing

19. Line 215 full stop missing

20. Line 244 details about the Abs are missing

21. Line 257 u shuld be µ

22. Line 266 px is missing

23. Line 860 the scale bar in figure is missing as well as a neuronal marker

24. Line 369-370 typos “ribosome in from”

25. Line 381 I guess that “Comparing” should be comparison

26. Line 382 CamkIIa and Shank3 should be in italic font

27. Line 391 I guess that the subject of the sentence is missing

28. Line 420 what do the authors mean by “contrasting cell types”?

29. Line 428: “pyramidal neuron differentiated translation candidates” please rephrase

30. Line 437 “cis” should be in italic font

31. Lines 451-552: the authors should mention that Sst and Cort are two of the differentially expressed genes resulting from their analysis, otherwise they look like two random examples

32. Line 503 Full stop missing

33. Lines 507-511: that statement on the cell specificity of the majority of the local candidates and the following comment completely lose their value given the statement in lines 517-518

34. Line 523: Have Gsr and Capn11 a specific known role in neuros that should be mentioned or are they just examples from the differential expression analysis?

Rev. #2.

In their article “Differences in local translatome across distinct neuron types is mediated by both baseline cellular differences and post-transcriptional mechanisms”, the authors present transcriptome sequencing data that surveys the RNAs engaged in translation in the synapses of two different neuronal cell types (pyramidal and GABAergic). This kind of data is termed “translatome” in the field.

With comparisons with controls (without enrichment for translated RNAs, cell type or synaptic localisation), and between cell types, the authors show that difference between synaptic translatomes are mainly driven by differences between transcription programs in each cell type, but that nevertheless some commonalities remain.

The authors also show evidence that there may be mechanisms acting on differential targeting to the synaptic translatome between cell types, as they found examples of RNAs expressed at the same levels in cell bodies but not enriched at the same level in synapses.

Among the RNAs found in the synaptic translatomes, the authors found some neuropeptide precursors, and validated synaptic localisation with in situ hybridisation and enrichment in the ribosomal fraction by qPCR.

Major comments

--------------

1) The resolution of the figures in the manuscript PDF was too low. In case of microscopy experiments, the low resolution made it impossible to properly assess the strength of the evidences provided by the authors (although it was still possible to understand what kind of evidence the authors wanted to show).

2) The authors wrote “As these regions could contain regulatory sequences that act as binding sites for RNABPs mediating localization or translational control, the 3'UTRs were analyzed for enriched novel motifs using MEME”. In the methods section, they write that “the longest available UTR sequence for each candidate was selected”. The main text explains that previous results showed that 3'UTRs were longer in the synaptic fraction, and then follows by concluding that the current study confirms the previous finding that 3'UTRs are enriched with certain motifs. This is circular reasoning, because the choice of 3'UTRs in this study was not driven by the presented data, but only by the assumption that they should be the longest, given what was previously published. Therefore, I think that the claim that the authors could “again find a poly-A binding motif enriched in somatic candidates and a motif that highly resembles a G-quadruplex in local candidates” is not sufficiently supported by the input data to this part of the analysis, which is only a gene list with no evidence on UTR length. The same applies to the paragraph detailing the AME analysis. (This might be a possible explanation to the fact that the authors did not see a difference of motif between the 3'UTRs of the two cell types in paragraph 436-445).

Minor comments

--------------

* The authors wrote “In order to identify candidate transcripts for local translation in each cell type, we applied an analysis to select for transcripts that are both enriched in the SNF and bound to ribosomes in from the cell type of interest”. While I understand that this approach aims at filtering for functionally relevant hits, I find the explanation misleading: if a RNA is bound to ribosomes in the synapse, but is either not enriched or even depleted in abundance when comparing whole cell to synapses, this does not rule out the possibility of local translation.

(side comment: note the “in from” typo in the authors text)

* The source code of the translatome data processing and analysis would be valuable to other researchers, and I encourage the authors to release it on appropriate platforms, such as Zenodo, Figshare, etc.
